# A 3D Platform to Investigate Dynamic Cell-to-Cell Interactions Between Tumor Cells and Mesenchymal Progenitors

**DOI:** 10.3389/fcell.2021.767253

**Published:** 2022-01-17

**Authors:** Giulia Golinelli, Rebecca Talami, Stella Frabetti, Olivia Candini, Giulia Grisendi, Carlotta Spano, Chiara Chiavelli, Gaëlle F. Arnaud, Giorgio Mari, Massimo Dominici

**Affiliations:** ^1^ Division of Oncology, Department of Medical and Surgical Sciences for Children and Adults, University-Hospital of Modena and Reggio Emilia, Modena, Italy; ^2^ Rigenerand Srl, Medolla, Modena, Italy; ^3^ Science and Technology Park for Medicine, Tecnopolo di Mirandola “Mario Veronesi”, Mirandola, Italy

**Keywords:** ewing’s sarcoma, 3D cell cultures, bioreactor, tumor-stroma interaction, mesenchymal stromal/stem cells, ddPCR, fluidic circuit, cancer therapy

## Abstract

We here investigated the dynamic cell-to-cell interactions between tumor and mesenchymal stromal/stem cells (MSCs) by the novel VITVO^Ⓡ^ 3D bioreactor that was customized to develop *in vivo*-like metastatic nodules of Ewing’s sarcoma (ES). MSCs are known to contribute to tumor microenvironment as cancer associated fibroblast (CAF) precursors and, for this reason, they have also been used as anti-cancer tools. Using dynamic conditions, the process of tissue colonization and formation of metastatic niches was recreated through tumor cell migration aiming to mimic ES development in patients. ES is an aggressive tumor representing the second most common malignant bone cancer in children and young adults. An urgent and unmet need exists for the development of novel treatment strategies to improve the outcomes of metastatic ES. The tumor-tropic ability of MSCs offers an alternative approach, in which these cells can be used as vehicles for the delivery of antitumor molecules, such as the proapoptotic TNF-related apoptosis inducing ligand (TRAIL). However, the therapeutic targeting of metastases remains challenging and the interaction occurring between tumor cells and MSCs has not yet been deeply investigated. Setting up *in vitro* and *in vivo* models to study this interaction is a prerequisite for novel approaches where MSCs affinity for tumor is optimized to ultimately increase their therapeutic efficacy. Here, VITVO^Ⓡ^ integrating a customized scaffold with an increased inter-fiber distance (VITVO50) was used to develop a dynamic model where MSCs and tumor nodules were evaluated under flow conditions. Colonization and interaction between cell populations were explored by droplet digital PCR (ddPCR). VITVO50 findings were then applied *in vivo*. An ES metastatic model was established in NSG mice and biodistribution of TRAIL-expressing MSCs in mice organs affected by metastases was investigated using a 4-plex ddPCR assay. VITVO^Ⓡ^ proved to be an easy handling and versatile bioreactor to develop *in vivo*-like tumor nodules and investigate dynamic cell-to-cell interactions with MSCs. The proposed fluidic system promises to facilitate the understanding of tumor-stroma interaction for the development of novel tumor targeting strategies, simplifying the analysis of *in vivo* data, and ultimately accelerating the progress towards the early clinical phase.

## Introduction

The process of tumorigenesis involves the acquisition of several genetic mutations that progressively lead cells to transformation, amplification, invasion, and metastasis (Mueller and Fusenig, 2004). All these steps are actively influenced by the tumor microenvironment (TME) (Wu and Dai, 2017). The TME comprehends a highly active stroma, in which the most represented cells are cancer-associated fibroblasts (CAFs) (Fiori et al., 2019; Piersma et al., 2020). Among the different sources of CAFs, mesenchymal stromal/stem cells (MSCs) are of great interest (Paunescu et al., 2011). The secretion of proinflammatory cytokines by tumor cells attracts MSCs into the tumor, as if it was “a wound that never heals” (Dvorak, 1986). In the tumor, MSCs acquire an activated phenotype which reminds that of CAFs and become part of the TME (Barcellos-de-Souza et al., 2016).

Ewing’s sarcoma (ES) is an aggressive tumor representing the second most common malignant bone cancer in children and young adults (Choi et al., 2014). Currently, an urgent and unmet need exists for the development of novel treatment strategies to improve the outcomes of metastatic ES (Grünewald et al., 2018; Casey et al., 2019). New avenues of research have been opened through the use of MSCs as vehicles for the delivery of antitumor molecules, such as the proapoptotic TNF-related apoptosis inducing ligand (TRAIL), against primary localized ES (Grisendi et al., 2015; Guiho et al., 2016). MSCs are considered as putative cells of origin for ES and display a tropism for tumor sites, as progenitors of the tumor supportive stroma (Dvorak, 1986; Lin et al., 2011). Despite MSCs tropism for tumors, the therapeutic targeting of metastases remains challenging and the interaction occurring between tumor cells and MSCs has not yet been deeply investigated (Kean et al., 2013). Setting up *in vitro* and *in vivo* models to study this interaction is a prerequisite for novel approaches where the MSCs affinity for the tumor and the retention inside the tumor microenvironment are ameliorated to ultimately increase the therapeutic efficacy (Golinelli et al., 2020; Golinelli et al., 2021).

It is well-known that bidimensional (2D) cell cultures do not allow to correctly recap physiological conditions that occur *in vivo* (Loessner et al., 2010; Ravi et al., 2017). *In vivo* models are the gold standard of pre-clinical studies, but they are highly expensive, time consuming and raise several ethical issues (Robinson et al., 2019). Three-dimensional (3D) *in vitro* models represent a good compromise between monolayered cell cultures and *in vivo* models (Marrella, 2021). Although spheroids are still one of the most used 3D systems in cancer research, they show some limitations. In spheroids, the TME is not reproduced and cells are somehow forced to interact with each other (Verjans et al., 2018). Further steps towards the development of more complex 3D models need to be taken. 3D systems recapitulating the extracellular environment support more complex cell-to-cell and cell-to-extracellular matrix (ECM) interactions and the combination with fluidic culture systems can better mimic several *in vivo* events like blood perfusion of tissues, some of the steps of the metastatic cascade and dynamic cell-to-cell interactions (Marrella, 2021). Compared with hydrogel-based systems, scaffolds created with synthetic biomaterials offer the advantage to precisely design the 3D architecture (Karami et al., 2019). As an example, Trachtenberg et al. developed a synthetic scaffold model of ES set in a fluidic system, which allowed to analyze how shear stress could influence cell proliferation and adhesion of tumor cells, recapitulating *in vivo*-like signals of tumors (Trachtenberg et al., 2018). However, very few studies exploring the use of hard porous system combined with fluidic in cancer research are reported in the literature. Taking all these considerations together, in this work a dynamic 3D co-culture system was fabricated, as a useful tool to better investigate the interactions occurring among tumor and MSCs.

VITVO^Ⓡ^ is a small and handy bioreactor specifically conceived to recreate *in vitro* an *in vivo*-like microenvironment. Cells can colonize the VITVO^Ⓡ^ matrix inner core which is formed by a fiber-based, synthetic, inert and biocompatible scaffold. VITVO^Ⓡ^ matrix allows cells retainment, offering mechanical support and favoring cell colonization and growth. The system is closed, directly monitorable thanks to the presence of two optical transparent oxygenation membranes and enables both static and dynamic application possibilities (Candini et al., 2019). Starting from VITVO^Ⓡ^ technology, a customized VITVO^Ⓡ^ bioreactor (VITVO50) integrating a different version of VITVO^Ⓡ^ matrix was produced. VITVO50 matrix has a wider inter-fiber distance compared with the one of VITVO^Ⓡ^, allowing to reduce cell entrapment and to set the right compromise between cell colonization and escape. Here, VITVO50 was used to develop a dynamic model which easily recapitulated the formation of *in vivo*-like ES metastatic nodules and enabled the study of tumor-MSCs interplay under flow conditions. Colonization and interaction between cell populations on VITVO50 were explored by droplet digital PCR (ddPCR). The use of ddPCR for the simultaneous amplification of different targets is progressively gaining importance in cancer research due to its high accuracy and sensitivity (Whale et al., 2016; Olmedillas-López et al., 2017). Setting up 3-plex ddPCR-based assays, we were able to detect both tumor cells and MSCs within the VITVO50 and evaluate the degree of interaction between tumor nodules and circulating MSCs. Finally, VITVO50 findings were applied *in vivo*. An ES metastatic model was established in NSG mice and the *in vivo* fate of TRAIL-expressing MSCs was investigated using a 4-plex ddPCR assay, looking at MSCs biodistribution in mice organs affected by metastases.

## Materials and Methods

### Cell Culture and Maintenance

The human ES cell line TC71 (RRID: CVCL_2,213, DSMZ, Braunschweig, Germany) was cultivated in Iscove’s modified Dulbecco’s medium (IMDM, Euroclone, Milan, Italy) supplemented with 10% FBS (Carlo Erba Reagents Srl, Cornaredo, Italy), 1% L-glutamine (200 mM; BioWhittaker, Lonza, Verviers, Belgium), and 1% penicillin-streptomycin (104 UI/ml penicillin and 10 mg streptomycin/ml; Carlo Erba Reagents Srl). Human adipose (AD)-MSCs were obtained as previously described from lipoaspirate specimens of individuals undergoing liposuction for esthetic purposes after approval by local Ethical Committee (Grisendi et al., 2010). After isolation, cells were grown in minimal essential medium with alpha modifications (*α*-MEM, Gibco, Thermo Fisher Scientific, Waltham, MA, United States) containing 2.5% platelet lysate (Macopharma, Tourcoing, France), 1% L-glutamine, 0.5% ciprofloxacin, and 0.2% heparin (Sigma-Aldrich, Saint Louis, Missouri, United States). Cells were incubated and maintained within a controlled atmosphere with 5% CO_2_ and a temperature of 37°C. The authentication of TC71 cell line was recently performed by the Leibniz Institute DSMZ - German Collection of Microorganisms and Cell Cultures GmbH.

### Viral Vectors and Cell Transduction

The TC71 ES line was engineered to express a red-shifted *Luciola italica* luciferase transgene using RediFect™ Red-FLuc-Puromycin Lentiviral Particles (PerkinElmer, Waltham, Massachusetts, United States), according to the manufacturer’s instructions. Photon emissions from the obtained luciferase-expressing TC71 (TC71 Luc) cells were measured using the IVIS Lumina XRMS (IVIS Lumina XRMS *In Vivo* Imaging System, PerkinElmer) with Living Image software (version 4.3.1, PerkinElmer), which was used to estimate the level of photon emission per cell. The MIGR1 vector, which encoded the red fluorescent protein DsRed, was used to stably transduce TC71 Luc cell line to facilitate the distinction of ES cells from MSCs on the VITVO50 matrix. Retrovirus production was performed using the FLYRD18 packaging cell line (RRID:CVCL_8871), as previously described (Marx et al., 1999; Grisendi et al., 2015).

MSCs were genetically modified with a pCCL PGK WPRE lentiviral vector encoding green fluorescent protein (GFP), as previously reported (Rossignoli et al., 2019).

### Fluorescence-Activated Cell Sorting

The expression of DsRed and GFP reporter genes by TC71 Luc and MSCs, respectively, were analyzed in phycoerythrin (PE) and fluorescein isothiocyanate (FITC) fluorescence channels. MSCs were further sorted by FACS to enrich for GFP + cells. Data were collected using a FACS Aria III flow cytometer (BD) and analyzed using FACS Diva software (BD).

### Design of a 3D Platform-Based Fluidic Circuit

**FIGURE 1:**
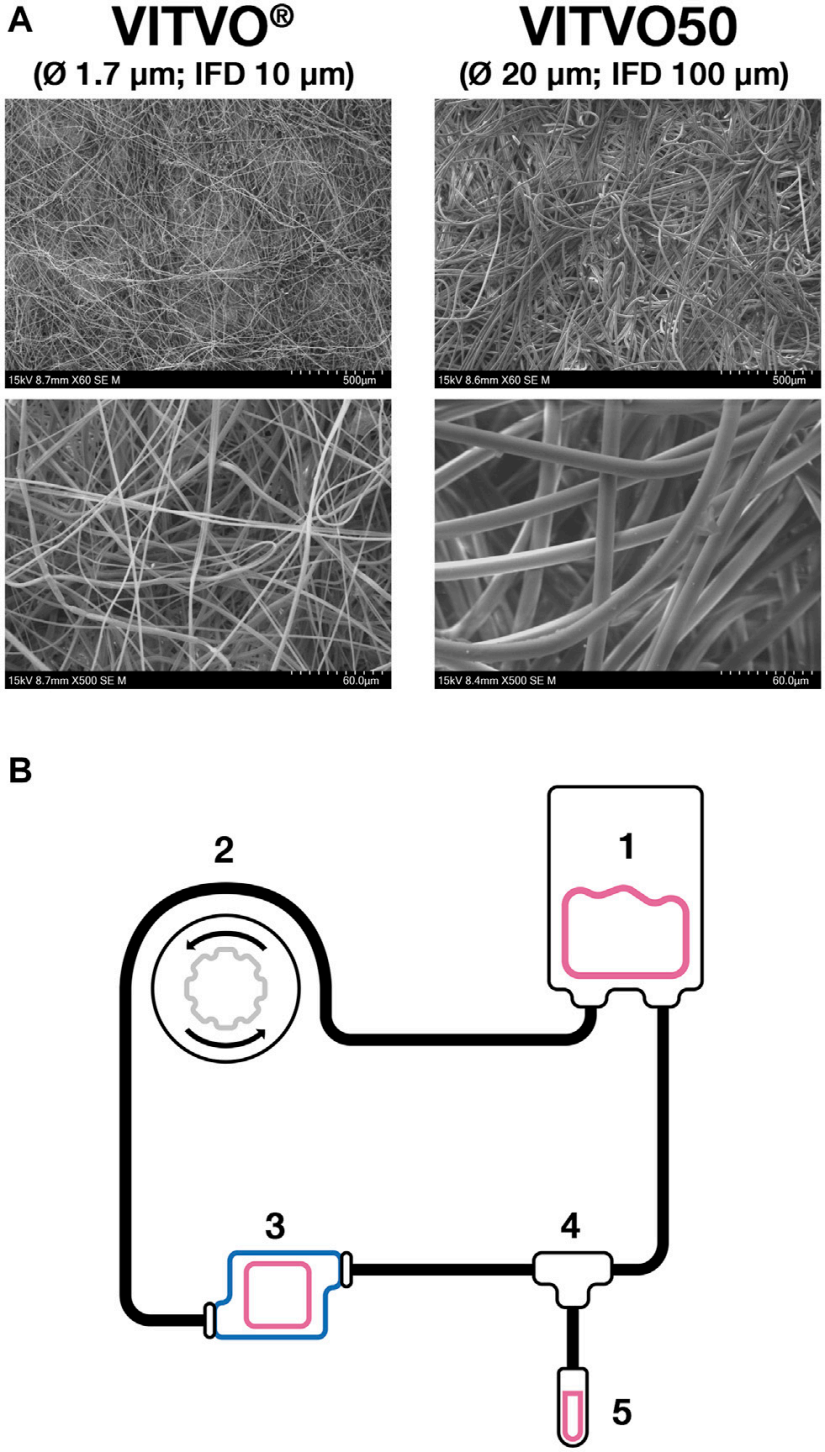
The VITVO50-based fluidic circuit. **(A)**, Representative images of both VITVO and VITVO50 inner matrices. VITVO^®^ matrix is composed by fibers with a diameter of 1.7 µm. The average inter-fiber distance (IFD) is 10 μm. VITVO50 matrix is characterized by larger fibers, with a diameter of 20 µm and an average IFD of 100 µm. Images were taken by scanning electron microscope TM4000 II plus 55E-0312 (Hitachi High-Technology Corporation, Tokyo, Japan). Original magnification ×60 for upper panels and X500 for lower panels. **(B)**, Schematic representation of the VITVO50-based fluidic circuit. A PVC bag (1) was loaded with medium and cells and then connected to a system of PVC tubes. The movement of the fluid was induced by an external peristaltic pump (2). The first PVC tube was equipped with two retaining stops that ensured correct tension once it is fitted into the pump rotor. The rotor compressed the tube, occluding part of it and therefore forcing the fluid within the circuit to move through. The first tube was connected to an extension tube that was in turn screwed to the inlet port of VITVO50 (3). The outlet port of VITVO50 was finally connected to a second extension tube, which was linked to the PVC reservoir bag to close the circuit. A valve (4) was plugged at the outlet port of the VITVO50 and could be handled to either force the fluid back to the reservoir bag or to an escape-collecting tube (5).

VITVO50 is a customized version of VITVO^Ⓡ^ bioreactor which integrates a matrix having a higher fiber diameter (Ø 20 µm in VITVO50 vs Ø 1.7 µm in VITVO^Ⓡ^) and an increased inter-fiber distance (100 µm in VITVO50 vs 10 µm in VITVO^Ⓡ^) (Figure 1A). VITVO50 was connected to a fluidic circuit built up as described in Figure 1B to generate a fluid-dynamic 3D model. Briefly, a PVC bag (Menny Medical, Menarini, Mirandola, Italy) was loaded with cell suspension in the medium and then connected to a PVC tube equipped with two retaining stops (Gilson, Middleton, Wisconsin, United States) which allow the tube securing to a peristaltic pump (MINIPLUS three Pump, Gilson). This tube was then connected to an extension PVC tube that was screwed to the inlet port of VITVO50 thanks to a luer-lock connection. The outlet port of VITVO50 was screwed to a second extension PVC tube which was connected to the reservoir bag to close the circuit. The cell flow was induced by the peristaltic pump integrated into the circuit. The retaining stop ensured correct tension of the tube that was fitted to the pump rotor. The rotor compressed the tube and occluded part of it. In this way, the fluid within the tube was forced to move through the circuit and enter VITVO50 from the inlet port. A valve (Menny Medical, Menarini, Mirandola, Italy) was plugged at the outlet port of the VITVO50 and could be handled to either force the fluid back to the bag or to an escape-collecting tube.

### Colonization of VITVO50 by Cell Perfusion Into the Fluidic Circuit

The VITVO50 bioreactor was used to recreate *in vivo*-like tumor nodules through the colonization of the 3D matrix under flow condition. The fluidic circuit was set up as described above. VITVO50 was primed by complete IMDM to prepare the inner matrix to host TC71 Luc cells. 1 × 10^6^ of TC71 Luc cells were diluted in 5 ml IMDM and transferred into the reservoir bag using a syringe. The bag was plugged in the circuit and the peristaltic pump was activated, forcing medium and cells through the circuit for an hour with a flow rate of 0.860 ml/min. Then, VITVO50 was removed from the circuit and the leftover fluid into the tubes was collected and centrifuged. The pellet was composed of cells that escaped the VITVO50 matrix (the escape) and remained in the circuit. The number of TC71 Luc cells colonizing the matrix was estimated by subtracting the escape from the starting number of tumor cells infused in the circuit. Colonization was microscopically verified by EVOS FL Auto microscope (ThermoFisher) looking at the DsRed fluorescence. VITVO50 was then incubated and maintained within a controlled atmosphere with 5% CO_2_ and a temperature of 37°C. After 24 h, VITVO50 colonization was further evaluated looking at TC71 Luc cell bioluminescence. The bioluminescent signal after luciferin (150 μg/ml; XenoLight D-Luciferin-K + Salt; PerkinElmer) administration was quantified by the GloMax^Ⓡ^ Discover plate reader (Promega, Madison, Wisconsin, United States) as a measure of tumor cell viability on the VITVO50 matrix.

Colonization of VITVO50 was also performed using MSCs. VITVO50 was primed by complete *α*-MEM. 1 × 10^6^ of MSCs were diluted in 5 ml *α*-MEM and transferred into the reservoir bag. The bag was plugged into the circuit and MSCs were perfused with a 0.860 ml/min flow rate for an hour, as for VITVO50 colonization with TC71 Luc cells. Similarly, the escape was counted to estimate the number of MSCs embedded within the VITVO50 matrix. Colonization was microscopically assessed by EVOS FL Auto microscope evaluating the GFP fluorescence.

### Interaction Between Tumor and MSCs in VITVO50 Under Flow Conditions

Once VITVO50 was colonized with the tumor, MSCs were introduced in the circuit and their interaction with tumor cells was evaluated under flow conditions. At 24 h from TC71 Luc cell colonization, 2.5 × 10^5^ MSCs were diluted in 5 ml of complete DMEM and transferred in the reservoir bag, which was then connected to the circuit to enable MSCs circulation and interaction with tumor cells under dynamic conditions. Perfusion of MSCs was performed for 5, 10 or 20 min with a flow rate of 0.860 ml/min. Then, VITVO50 was removed from the circuit and the escape was collected. The number of MSCs retained by the matrix was estimated subtracting the escape from the starting number of MSCs infused in the circuit. The retention of MSCs on the VITVO50 previously colonized by the tumor was also verified by EVOS FL Auto microscope observing both the GFP and the DsRed fluorescence. In an opposite setting, VITVO50 was colonized with MSCs and the interaction with floating tumor cells was assessed. 2.5 × 10^5^ TC71 Luc cells were diluted in 5 ml of complete DMEM and perfused into the circuit for 20 min as described above. The number of TC71 Luc cells which interacted with MSCs or bounded to the matrix fibers within the VITVO50 was estimated by escape counting. The retention of floating tumor cells on VITVO50 was also confirmed using EVOS FL Auto microscope. After interaction, all VITVO50 matrices were then preserved for molecular evaluation by ddPCR.

### Mouse Tissues

Mouse tissues were obtained from a previous mouse experiment conducted in accordance with the institutional and national guidelines and under approved protocols by the Local Ethical Committee on Animal Experimentation and by the Italian Ministry of Health (authorization no: 22/2020 PR). An animal model of metastatic ES was established in NSG (NOD.Cg-PrkdcSCID Il2rgtm1Wjl/SzJ, RRID: BCBC_4142) mice. The animals were intravenously (i.v.) inoculated with 2 × 10^6^ TC71 Luc cells suspended in 150 µl phosphate-buffered saline (PBS). Four days after tumor cell inoculation, the animals received multiple (*n* = 3) i. v. injections consisting of 1 × 10^6^ of MSCs delivering a soluble variant of TRAIL (sTRAIL MSCs), which were administered every 3 days in 150 µl PBS. Control mice was treated with control MSCs expressing the GFP. For the third injection, the MSCs were labelled with XenoLight DiR (8 μM; Perkin Elmer), following the manufacturer’s instructions. Animals were recurrently imaged using the IVIS Lumina XRMS (Perkin Elmer) to detect the bioluminescent signal from TC71 Luc cells and the DiR fluorescence of MSCs. Fixed regions of interest (ROIs) were drawn around the lungs, liver, and full body, and the total radiant efficiency [p/s/cm^2^/sr]/[µW]/cm^2^] of DiR fluorescence was quantified using Living Image software (Perkin Elmer). To compare mouse groups in terms of the relative distribution of MSCs in the lungs and liver, the total radiant efficiency of the lungs (or liver) was normalized against that for the full body, and the ratio was expressed as a percentage. The median and interquartile range (IQR) values were calculated and compared between groups. After 13 days, animals were sacrificed by the intraperitoneal injection of Tanax (Intervet Italia Srl, Italy), and the organs (lung, liver, and femur) were examined for the presence of metastases using IVIS. Organs were then preserved for molecular evaluation by ddPCR.

### Genomic DNA Extraction From VITVO50 and Mouse Tissues

After tumor-MSCs interaction, VITVO50 were kept inside the incubator for 3 h. Then, VITVO50 matrix was cut into pieces, placed into gentleMACS M Tubes (Miltenyi, Bergisch Gladbach, North Rhine-Westphalia, Germany) and homogenized in Tissue Lysis Buffer (TLA; Promega) using gentleMACS Dissociators (Miltenyi). After centrifugation at 200 *g* for 2 min, the homogenate was collected and incubated with proteinase K (2 mg/ml; Promega) at 56°C for 30 min. The lysate was carefully mixed and a representative fraction was sampled for genomic DNA (gDNA) extraction. gDNA was automatically isolated using a Maxwell 16 Instrument (Promega) using the Maxwell 16 LEV Blood DNA Kit (Promega), according to the manufacturer’s instructions. Contaminating RNA was removed by the addition of RNase (20 μg/ml; Promega) to the elution buffer. The gDNA concentration was measured using the Infinite M Nano (Tecan, Männedorf, Zürich, Switzerland), and gDNA samples were diluted to approximately 12.5 ng/μl. Mouse lungs and livers were explanted, maintained on dry ice, and stored at -80°C. For gDNA extraction from frozen organs, the above protocol set on VITVO50 matrix was performed. Finally, gDNA samples were diluted to approximately 40 ng/μl.

### Droplet Digital PCR

**FIGURE 2:**
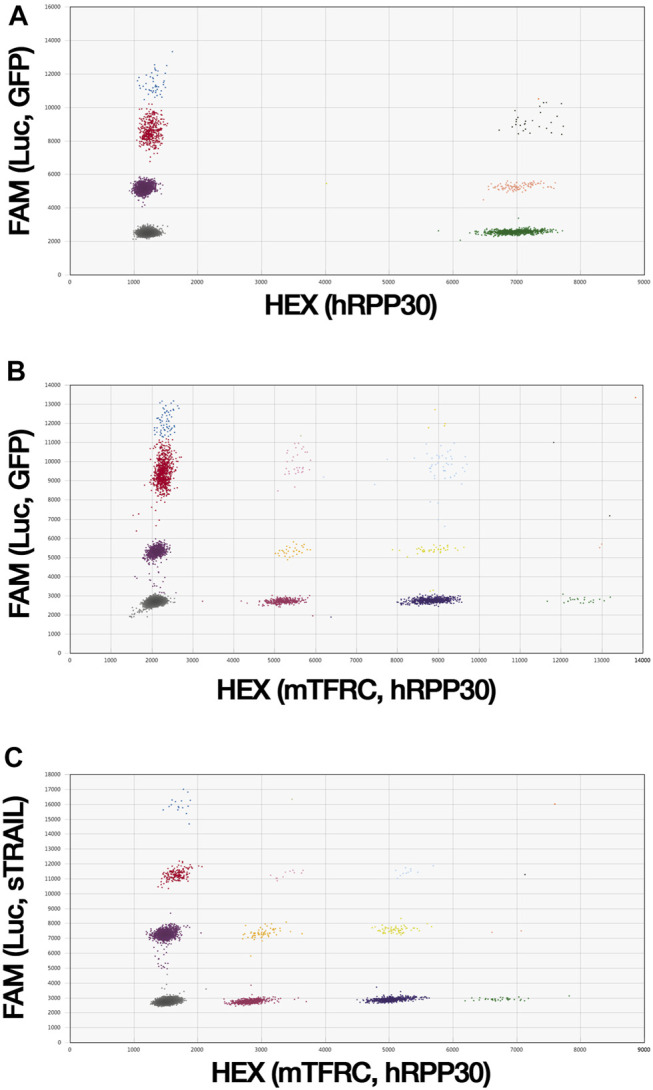
ddPCR assays are able to simultaneously detect tumor cells and gene-modified MSCs. **(A)**, A 3-plex ddPCR assay was developed to quantitively evaluate tumor cells and MSCs in the VITVO50. Custom primers and probes were designed on Luc and GFP target genes to identify tumor cells and MSCs, respectively. hRPP30 was used as reference gene to estimate the total number of human cells. Due to the DNA distribution within the droplets, in the 3-plex ddPCR assay droplets were sorted out in eight different colored clusters. **(B)** and **(C)**, 4-plex ddPCR assays were set up to simultaneously investigate MSC biodistribution and antitumor effect in the ES metastatic model. gDNA was obtained from lungs and liver of mice. Because of the random distribution of DNA into the droplets, in 4-plex ddPCR assays droplets are clustered into sixteen different colored groups. The control 4-plex ddPCR assay **(B)** was developed combining Luc and GFP target assays with hRPP30 and mTfrc reference assays to detect human cells, TC71 Luc cells and control MSCs, localized in mice organs. The sTRAIL 4-plex ddPCR assay **(C)** was set up matching Luc and sTRAIL target assays with the mTfrc and hRPP30 reference assays to distinguish between TC71 Luc tumor cells and effector MSCs in mice organs.

Custom primers and probes conjugated to 6-carboxyfluorescein (FAM) were designed to target selected genes using the Primer3Plus Web interface (Supplementary Table S1) and produced by Bio-Rad (Hercules, California, United States). The Luc assay was designed to target the luciferase gene and was used to detect TC71 Luc tumor cells. The GFP assay was designed to target the GFP gene for the detection of control MSCs. The sTRAIL assay was designed to target the sTRAIL gene and was used to assess sTRAIL MSCs. The human ribonuclease P/MRP subunit P30 gene (hRPP30; AssayID: dHsaCP2500350, Bio-Rad) and the *Mus musculus* transferrin receptor gene (mTfrc; AssayID: dMmuCNS420644255, Bio-Rad) reference assays, which were both conjugated with hexachlorofluorescein (HEX), were used for human and murine cell detection, respectively, and for gene copy number (CN) analysis. The absolute quantification of each target and reference gene was performed using the QX200 ddPCR System (Bio-Rad). Briefly, the ddPCR mixture was composed of extracted gDNA, the target and reference primers and probes, 4× ddPCR Multiplex Supermix for Probes (Bio-Rad), DTT (4 mM; Thermo Fisher), and nuclease-free water (Thermo Fisher). The ddPCR reaction mixture was partitioned into droplets and cycled with the following conditions: 95°C for 10 min (1 cycle); 94°C for 30 s and 55°C for 2 min (37 cycles), with a ramp of 2°C/s; 98°C for 10 min (1 cycle), and 4°C hold for at least 2 h. Data analysis was performed by QuantaSoft Software (Version 1.5.38, Bio-Rad). Positive droplets containing amplification products were discriminated from negative droplets without amplification products. The concentration (copies/µl) of each target and reference gene was reported automatically by QuantaSoft Software. gDNAs isolated from TC71 Luc cells and gene-modified MSCs were first used to estimate the CN of the target genes using 2-plex ddPCR assays in which the assay for the target gene is combined with the hRPP30 reference assay, at a final concentration of 900 nM primers/250 nM probe. The ratio between the target and reference concentrations was multiplied by the reference CN per genome (two for diploid genomes) to calculate the target gene CN. To analyze tumor-MSCs interaction on VITVO50 matrix under flow conditions, 3-plex ddPCR assays were carried out on VITVO50-derived gDNA (Figure 2A). gDNA was isolated as described above and 25 ng were used for the ddPCR reaction. The 3-plex ddPCR assay combined Luc (270 nM primers/75 nM probe) and GFP (1,125 nM primers/312.5 nM probe) assays with hRPP30 (900 nM primers/250 nM probe) reference assay and allowed to simultaneously detect the presence of TC71 Luc tumor cells and MSCs. Gene concentration was divided by the specific gene CN to calculate the number of TC71 Luc cells and of MSCs, as well as the total number of human cells per μl of ddPCR reaction. Ratios of the number of TC71 Luc cells (or of MSCs) per μl to the total number of cells per μl were calculated and expressed as percentage. Ratios between the numbers of MSCs (or of TC71 Luc cells) and TC71 Luc cells (or MSCs) were also obtained.

For the analysis of the ES metastatic model, 4-plex ddPCR assays were performed on organ-derived gDNAs to simultaneously detect the presence of TC71 Luc tumor cells, gene-modified MSCs, and murine cells (Figures 2B,C). The control 4-plex ddPCR assay combined Luc (270 nM primers/75 nM probe) and GFP (1,125 nM primers/312.5 nM probe) target assays with mTfrc and hRPP30 reference assays (both at 900 nM primers/250 nM probe). In the sTRAIL 4-plex ddPCR assay, Luc (765 nM primers/212.5 nM probe) and sTRAIL (900 nM primers/250 nM probe) target assays were combined with the mTfrc (765 nM primers/212.5 nM probe) and hRPP30 (900 nM primers/250 nM probe) reference assays. The target and reference gene concentrations were divided by the respective gene CNs to calculate the numbers of TC71 Luc cells and gene-modified MSCs and the numbers of human and murine cells per µl of the ddPCR reaction. The total number of cells per µl was obtained by combining the human and murine cell numbers. Ratios between the number of TC71 Luc cells (or gene-modified MSCs) per µl and the total number of cells per µl were calculated. For each mouse group, the median (IQR) values were derived and multiplied by 1,000, and the groups were then compared in terms of metastases growth and MSCs distribution. Ratios between the numbers of gene-modified MSCs and TC71 Luc cells in both the lungs and liver were also obtained for each group.

### Statistics

VITVO50 data are expressed as the mean ± standard deviation (SD). A two-tailed *p*-value of <0.05, assessed using Student’s t-test, was considered significant. Each experimental group was assayed at least in triplicate. Analyses were performed using Excel 2020 (Microsoft Inc., Redmond, WA, United States). *In vivo* data were reported as the median and IQR. Comparisons between different experimental groups were performed by using the Wilcoxon–Mann–Whitney test, whereas comparisons between the lungs and liver within the same experimental group were performed using the Wilcoxon signed-rank test for paired data. The analyses were performed with R, 3.4.3, statistical software (The R Foundation for Statistical Computing, Wien, Austria). All tests were two-tailed, and the confidence level was 95% (*p* < 0.05).

## Results

### TC71 Cells and MSCs Effectively Express Reporter Genes

**FIGURE 3:**
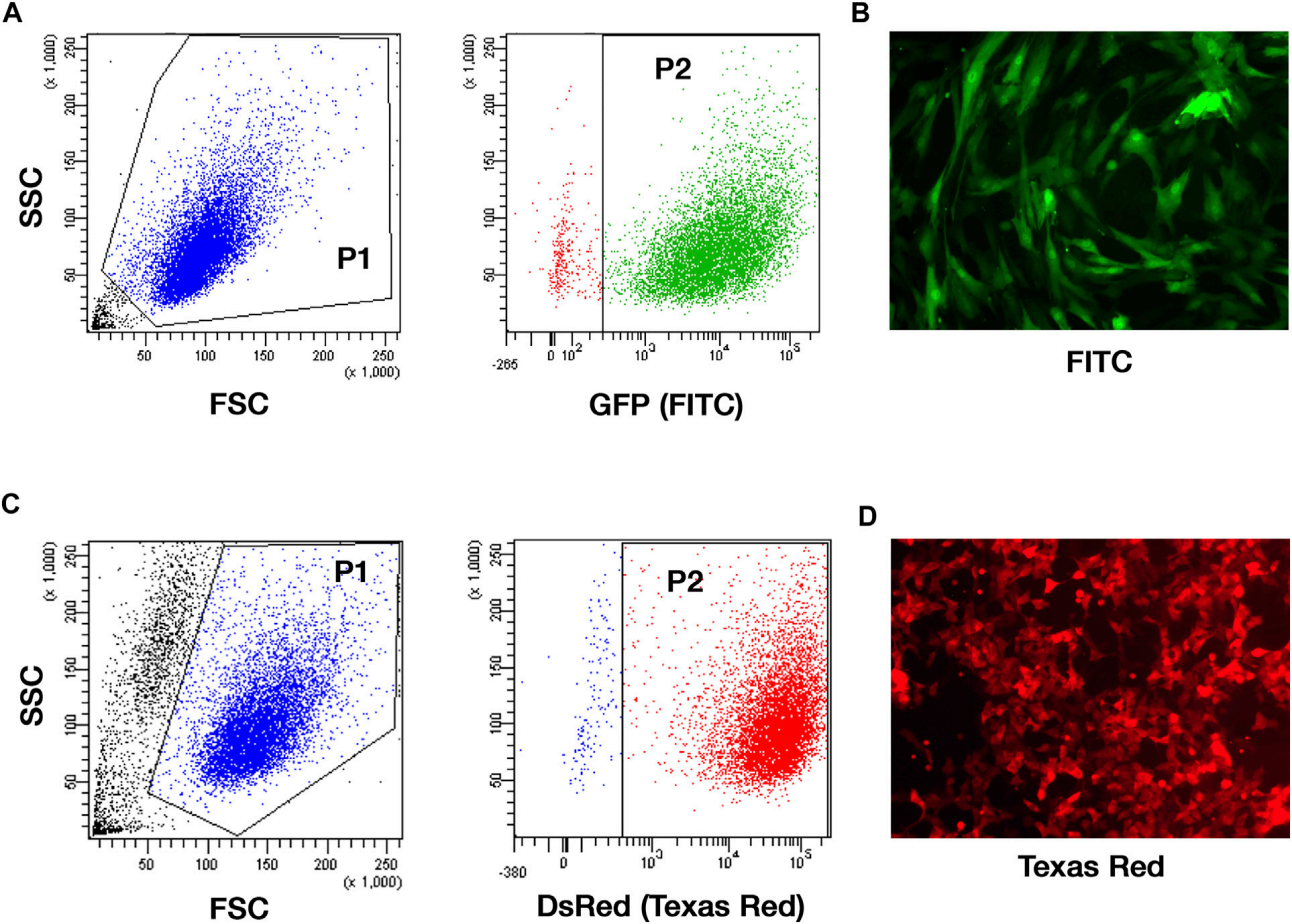
TC71 cell line and MSCs effectively express reporter genes. **(A)**, Gating strategy for the analysis of the GFP expression on MSCs transduced with GFP-encoding pCCL PGK WPRE lentiviral vector and sorted by FACS. Gate 1: forward scatter (FSC) and side scatter (SSC) area was used to enrich for intact cells (P1). Gate 2: GFP fluorescence recorded in the logarithmic scale was used to identify MSC GFP+ (P2; 93.9 ± 0.4%). **(B)**, Representative picture of GFP-expressing MSCs by fluorescence microscopy (original magnification ×100). **(C)**, Gating strategy for the analysis of the DsRed expression on TC71 Luc cells transduced with DsRed-encoding MIGR1 vector. Gate 1: FSC and SSC area was used to enrich for intact cells (P1). Gate 2: DsRed fluorescence assessed in the logarithmic scale allowed the identification of DsRed-expressing TC71 Luc cells (P2; 98.5 ± 0.7%). **(D)**, Representative picture of DsRed-expressing TC71 Luc cells by fluorescence microscopy (original magnification ×100).

MSCs were genetically modified for the expression of the GFP and sorted by FACS to finally obtain 93.9 ± 0.4% of GFP + cells (Figures 3A,B). TC71 cells have been engineered to express the red-shifted *Luciola italica* luciferase transgene and the obtained TC71 Luc cells were tested by IVIS, reporting a photon emission of 1774 ± 582 p/s per cell (not shown). To better investigate tumor-MSCs interaction in VITVO50, TC71 Luc were further modified to express the DsRed (98.5% of DsRed-expressing cells by FACS; Figures 3C,D). By these different labelling strategies, MSCs and TC71 cells were clearly distinguishable on VITVO50 looking at GFP and DsRed fluorescence.

Custom primers and probes were designed on unique target genes that derived from exogenous viral infections (Luc and GFP), while reference assay was selected to detect the *Homo sapiens* hRPP30 gene. Then, we set up 2-plex ddPCR assays that simultaneously amplified a target gene with the human reference gene to determine copy numbers of Luc and GFP target genes. In details, 2-plex ddPCR assays reported 4.86 ± 0.4 GFP copies for MSCs and 4.43 ± 0.08 Luc copies for TC71 Luc cells (data not shown).

### Establishment of in Vivo-like ES Nodules in VITVO50 Bioreactor

**FIGURE 4:**
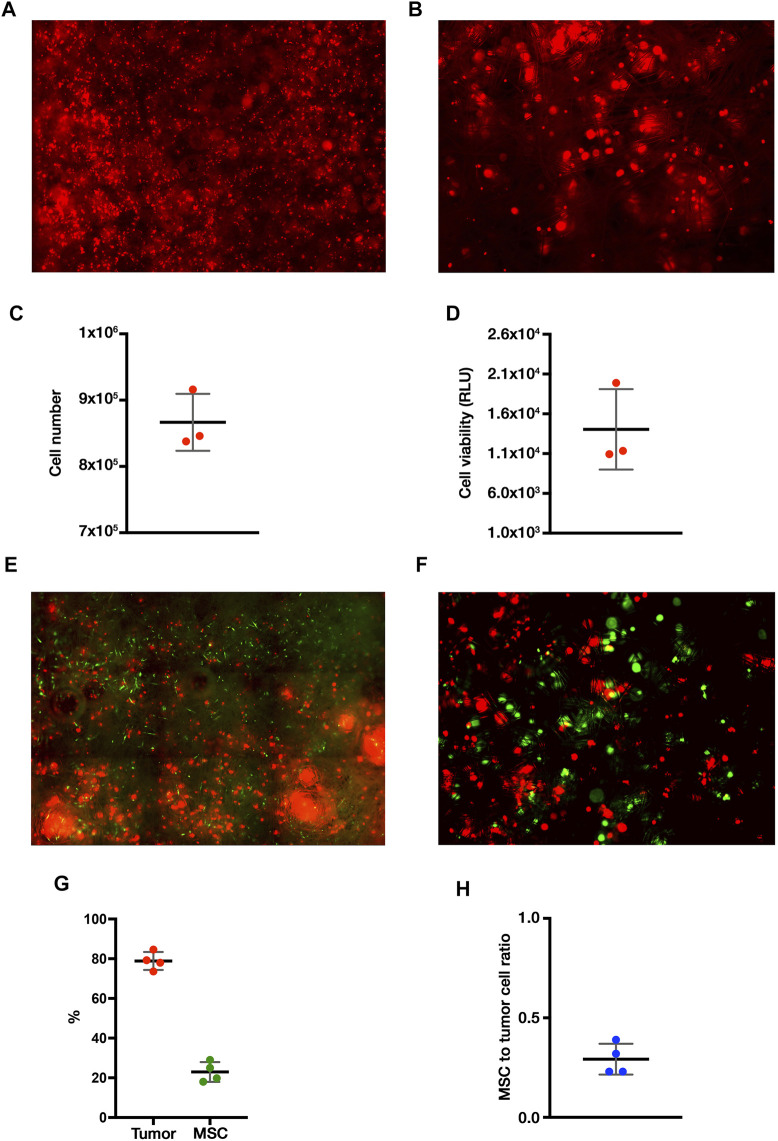
The VITVO50-based fluidic circuit allows to investigate dynamic cell-to-cell interactions between tumor nodules and MSCs. **(A)**, Scan of nine representative fields of the VITVO50 matrix colonized by TC71 Luc cells (red; Objective 4×). **(B)**, Representative picture taken at a higher magnification showing the formation of in vivo-like nodules of tumor cells in the VITVO50 matrix (red cell aggregates; Objective 10×) **(C)** Mean (SD) number of tumor cells colonizing VITVO50 bioreactor derived from escape data. Numbers of independent experiments *n* = 3. **(D)**, Bioluminescent signal after luciferin administration was used to quantify the viability of TC71 Luc cells seeded on the matrix. Data are expressed as the mean (SD). Numbers of independent experiments *n* = 3. **(E)**, Scan of nine representative fields of the VITVO50 matrix to evaluate interaction between TC71 Luc cells (red) and MSCs (green; Objective 4×). **(F)**, Representative picture taken at a higher magnification showing MSCs trapped between the fibers of VITVO50 matrix and interacting with tumor nodules (Objective 10×). **(G)**, Mean (SD) of the percentage of tumor cells (red) and MSCs (green) within the VITVO50 matrix obtained using the 3-plex ddPCR assay. Numbers of independent experiments n = 4. **(H)**, Ratio between the number of MSCs and tumor cells on VITVO50 matrix expressed as mean (SD). Numbers of independent experiments *n* = 4.

The VITVO50-based fluidic circuit allowed us to model the process of tissue colonization and creation of metastatic nodules by migrating tumor cells, mimicking ES dynamic in patients. The circuit was assembled as described in materials and methods. Preliminary tests were carried out to determine the best colonization condition, which is characterized by the uniform distribution of tumor cells within the VITVO50 matrix, with the absence of major cell clumps (data not shown). For the final setting, we set a colonization of VITVO50 with 1 × 10^6^ TC71 Luc cells for an hour. A flow rate of 0.860 ml/min was selected as sufficiently slow to allow cells to adhere to the fibers, but fast enough to prevent the formation of cell clumps around the inlet port of VITVO50. Three VITVO50 were parallelly colonized under flow. VITVO50 were then removed from the circuit and observed under the microscope (Figure 4A). VITVO50 colonization was sufficiently homogeneous. Cells were randomly attached between the matrix fibers, arranging themselves through the whole thickness of the matrix where they created small, dispersed nodules (Figure 4B). Quantitative data were further collected by escape counting and cell viability analysis on VITVO50. The average number of tumor cells that escape after colonization was 143,416.7 ± 25,807.4. The VITVO50 were therefore colonized by 866,583.3 ± 42,994.4 tumor cells (Figure 4C). After 24 h, bioluminescence was collected as a measure of tumor cell viability on VITVO50 (Figure 4D). Escape and bioluminescence data were coherent; as an example, tumor cell viability was slightly higher on the third VITVO50 which had the lower escape rate of 114,000 cells. Collectively, these tests allowed us to define the fluidic parameters to establish in vivo-like ES nodules within VITVO50. This VITVO50-based model can be employed to analyze interactions between tumor cells and MSCs under flow conditions.

### MSCs Interact With the in Vivo-like ES Nodules in VITVO50 Under Flow Conditions

VITVO50 (*n* = 3) previously colonized by TC71 Luc cells were employed to define an optimal interaction time between tumor cells and MSCs. In detail, 5, 10, and 20 min-interaction times were tested to allow MSCs circulation for about once, twice, and three times, through the device, respectively. When the interaction was over, the VITVO50 was unplugged from the circuit and cells were observed by fluorescence microscopy. We showed that perfused MSCs homogenously localized through the entire matrix (Figure 4E). At a higher magnification, MSCs appeared to bind to the fibers within the matrix and, most importantly, they seemed to interact with tumor nodules (Figure 4F). Looking at the escape, the percentage of perfused MSCs that bind to VITVO50 were as follows: 3.6, 32.4 and 34.1% after 5, 10, and 20 min of interaction, respectively (data not shown). The ratio of the number of tumor cells or MSCs to the total number of cells inside VITVO50 was calculated from escape data and expressed as percentage (Table 1). The MSCs to tumor cell ratios were as follows: 0.01, 0.1 and 0.1, for the interaction times of 5, 10 and 20 min, respectively. VITVO50 were left in incubator for 3 h, then gDNA was extracted from the VITVO50 matrix. A 3-plex ddPCR assay was performed to analyze tumor-MSCs interaction more accurately through the simultaneous detection of TC71 Luc cells and MSCs in the VITVO50. Starting from ddPCR data, percentages of tumor cells and MSCs as well as MSCs to tumor cells ratios were calculated (Table 1). VITVO50 (5 min) reported 6.2% of MSCs and 92.4% of TC71 Luc cells. The MSCs to tumor cell ratio was 0.1. As for VITVO50 (10 min), ddPCR showed 14.7% of MSCs and 88.3% of TC71 Luc cells, having a MSC to tumor cell ratio of 0.2. Finally, VITVO50 (20 min) was colonized by 23.7% of MSCs and 78.5% of TC71 Luc cells, with a MSC to tumor cell ratio of 0.3. Compared with escape data, ddPCR reported a higher retention of MSCs in the VITVO50, especially for the VITVO50 (20 min; Table 1). During interaction under flow, we hypothesize that few tumor cells could have detached from the matrix and contributed to the escape, therefore underestimating the percentage of retained MSCs. In support, TC71 is a highly metastatic cell line that by itself grows in semi-adherence conditions and does not establish strong interaction with the support. To confirm this hypothesis, we built up a second circuit, in which the classic VITVO^Ⓡ^ was used as a sort of sift (not shown). Given its low inter-fiber distance, VITVO^Ⓡ^ was able to trap escaped cells on the side of the matrix close to the inlet port, whether they were MSCs or tumor cells. After 20 min of interaction, we oriented the valve at the outlet port of VITVO50 to make the medium flow for 10 min through the classic VITVO^Ⓡ^, installed downstream of the VITVO50. By microscope analysis of VITVO^Ⓡ^, we observed the presence of few tumor cells that had detached from VITVO50, together with escaped MSCs (Supplementary Figure S1). This confirmed that relying only on escape to study interactions between TC71 cells and MSCs in the VITVO50 is not correct, as it does not allow to distinguish between the two cell types. Despite being more time consuming, ddPCR is far more accurate to study dynamic cell-to-cell interactions in the VITVO50-based fluidic circuit. As an alternative, flow cytometry could be employed to discriminate between tumor cells and MSCs escaped from VITVO50 matrix during interaction.

**TABLE 1:** Comparison between escape-derived data and ddPCR-derived ones on the tumor-MSCs interplay after 5, 10 and 20 min-interaction times.

	Escape data (%)		ddPCR data (%)	
Time (minutes)	Tumor	MSCs	Tumor	MSCs
5	99.0	1.0	92.4	6.2
10	91.2	8.8	88.3	14.7
20	90.8	9.2	78.5	23.7

The interaction time of 20 min allowed a fine retention of MSCs within VITVO50 and was selected as the optimal. During 20 min of flow, MSCs can pass through the device for approximately three times, thereby fostering the interaction with tumor cells. Additionally, only a portion of MSCs had been trapped into the matrix of VITVO50. As it does not lead to total MSCs retention, the proposed fluidic setting could be applied to study interactions between tumor cells and MSCs, where tumor affinity of MSCs has been increased by novel targeting strategies (Golinelli et al., 2020; Golinelli et al., 2021).

### Evaluating the Reproducibility of Our VITVO50-Based Fluidic Model

Colonization of VITVO50 with 1 × 10^6^ tumor cells and perfusion of 2.5 × 10^5^ MSCs for 20 min was selected as the best setting to study cell-to-cell interaction on VITVO50. Using this setting, we next confirmed the reproducibility of our dynamic interaction model. Four dynamic VITVO50-based fluidic circuits were set up as described in materials and methods. ES nodules were established in the VITVO50 matrix, then MSCs were perfused and left in interaction under flow for 20 min. After 3 h, gDNA was extracted from the matrix and 3-plex ddPCR assay was performed. On average, the four VITVO50 were colonized for the 78.9 ± 4.5% by the tumor and the remaining 22.9 ± 5.0% was represented by interacting MSCs that have been retained on the matrix (Figure 4G). The average MSCs to tumor cell ratio was 0.3 ± 0.1 (Figure 4H). Collectively, we confirmed the reproducibility of the dynamic VITVO50-based model, as no significant difference in both colonization by the tumor and retention of MSCs was detected. Setting up a VITVO50-based fluidic circuit we were able to investigate dynamic cell-to-cell interactions between tumor nodules and MSCs. Finally, 3-plex ddPCR assay proved to be a sensitive method to study colonization and interaction within VITVO50.

### Perfused MSCs Effectively Engrafted to the Lungs and Liver in an ES Metastatic Model

**FIGURE 5:**
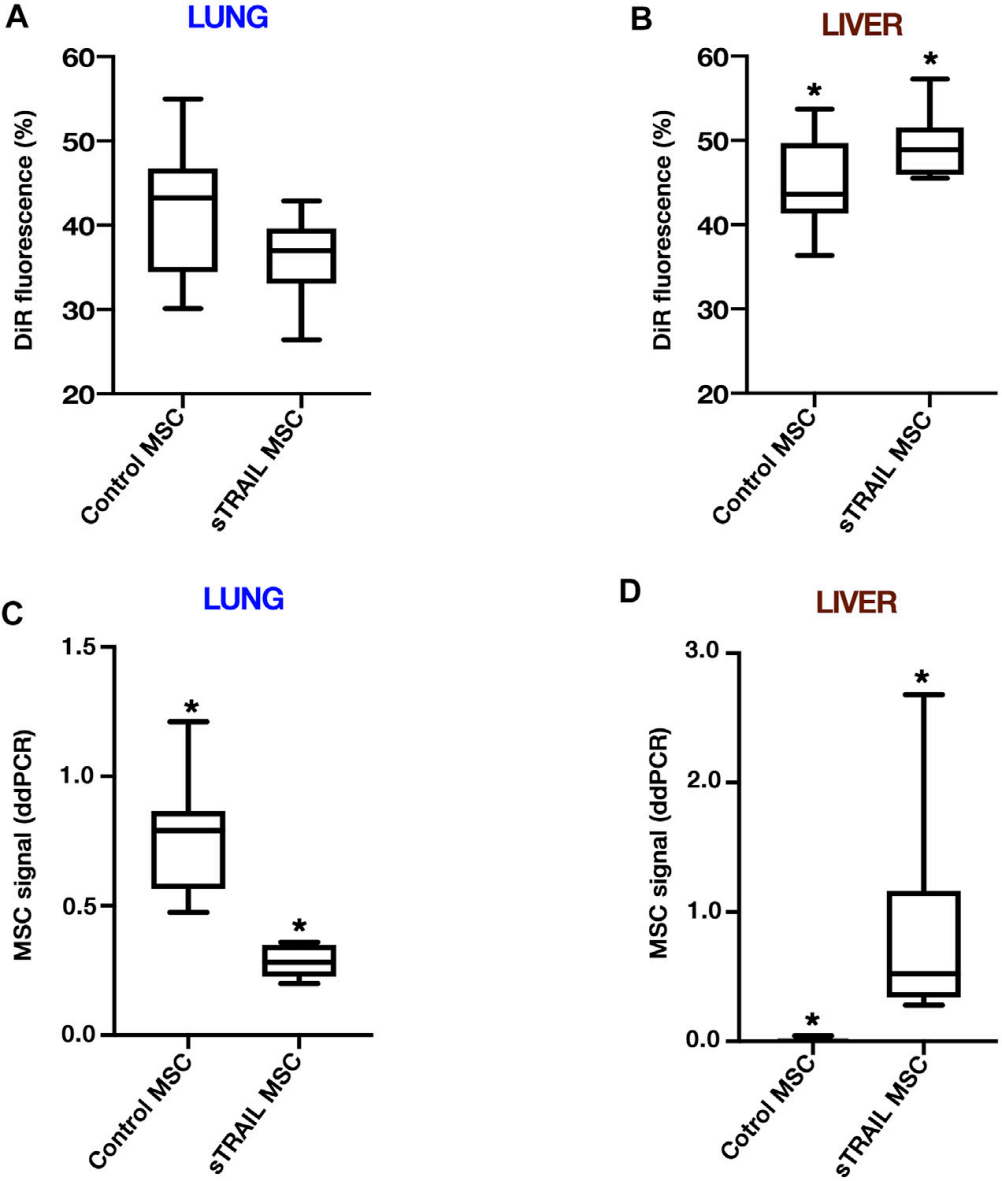
MSCs effectively engraft to the lungs and liver of ES metastatic model mice. **(A,B)**, The final dose of gene-modified MSCs was labelled by DiR dye before the infusion to investigate the MSCs biodistribution and tumor-targeting efficiency. DiR-labelled MSCs were followed for 2 days after intravenous administration by IVIS. DiR signals in lungs and liver were normalized to full body DiR fluorescence. The ratios for the lungs **(A)** or liver **(B)** were expressed as a percentage and median (interquartile range; IQR) values were calculated. Box plots show the data for an early time point of 4 h after MSCs infusion. For the liver, *p < 0.05. **(C,D)**, Three days after MSCs infusion, MSCs engraftment was confirmed on extracted organs by control or sTRAIL 4-plex ddPCR assays. Ratios of the number of gene-modified MSCs per µl to the total number of cells per µl were calculated. The median (IQR) values were derived and multiplied by 1,000, and groups were then compared in terms of MSCs distribution in lungs **(C)** and liver **(D)**. For the lungs, *p < 0.001. For the liver, *p < 0.001. All *p*-values have been calculated using the Wilcoxon–Mann–Whitney test.

VITVO50 findings were finally applied for the analysis of archived specimens of a previous experiment conducted *in vivo*. An ES metastatic model was established in NSG mice using TC71 Luc cells. Primary organs affected by metastases were lungs and liver, with a tumor cell quantity in the liver which was significantly higher than that in the lungs (*p* < 0.005; not shown). Biodistribution of both control MSCs and sTRAIL MSCs after i. v. injection was investigated. DiR-labelling of the last MSCs dose enabled us to track MSCs fate *in vivo* early after infusion. At 4 h post MSCs injection, fluorescence was equally distributed between lung and liver in control MSCs group, while in sTRAIL MSCs group, the hepatic signal was higher than the pulmonary one (*p* < 0.05; Figure 5). In the lung, the sTRAIL MSCs signal was lower than that for control MSCs, though not significant (*p* > 0.05; Figure 5A), whereas in the liver we observed an opposite trend (*p* < 0.05; Figure 5B). After 48 h, the sTRAIL MSC fluorescence was still lower in the lungs and higher in the liver compared with that in the control MSC group (not shown). At 3 days after MSC infusion, the DiR signal becomes less specific due to the described microenvironmental contamination (Lassailly et al., 2010). Taking this into account, MSC engraftment was confirmed in lungs and livers using sensitive 4-plex ddPCR assays, as described in materials and methods. ddPCR confirmed that the retention of control MSCs in the lungs was significantly higher than the retention of sTRAIL MSCs (*p* < 0.05; Figure 5C). Compared with the lungs, higher numbers of sTRAIL MSCs were detected in the liver (2.0-fold change, *p* < 0.05), whereas control MSCs were nearly undetectable (Figure 5D). Examining the MSCs to tumor cell ratio in the lungs, no differences were observed among the two groups (*p* > 0.05), and ratios were as follows: 5.774 (3.522–8.229) for the control MSCs group, and 7.674 (4.563–21.868) for the sTRAIL MSCs group. In the liver, the ratio was 0.000 for control MSCs group, as MSCs were detected at extremely low levels in only a few mice. As for sTRAIL MSCs group, tumor cells outnumber MSCs in the liver, resulting in a MSCs to tumor cell ratio of 0.466 (0.099–1.002). Overall, ddPCR data confirmed that MSCs were able to effectively engraft to the lungs and liver of ES model mice. In the sTRAIL group, lung metastases were strongly reduced compared with control MSCs group (not shown). However, the number of MSCs engrafted to the lung varied accordingly, maintaining a relatively constant MSCs to tumor cell ratio for both groups. Of notice, in the liver, the ratio between sTRAIL MSCs and tumor cell was similar to the one obtained in the VITVO50-based fluidic circuit for the study of interaction between floating MSCs and in vivo-like ES nodules.

### ES Colonized a CAF-like Environment Made by MSCs to Be Targeted in the VITVO50

**FIGURE 6:**
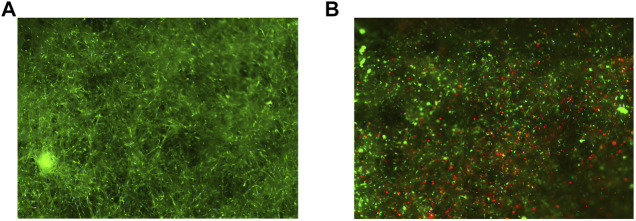
The VITVO50-based fluidic circuit can be employed to mimic the process of tumor colonization of a CAF-like microenvironment. **(A)**, Scan of nine representative fields of the VITVO50 matrix colonized by MSCs (green; Objective 4×). **(B)**, Scan of nine representative fields showing tumor cells (red) trapped into the VITVO50 matrix as a result of interactions they established with MSCs and with the fibers. Objective 4×.

We further assessed whether the VITVO50-based fluidic circuit could be a feasible tool to study cell-to-cell interactions in an opposite setting compared to the previous one. Here, VITVO50 (*n* = 3) was first colonized by MSCs, aiming to recreate a CAF-like microenvironment (Figure 6A). Tumor cells were then infused in the circuit, and we investigated their migration and retention in the VITVO50 matrix, as a result of interactions they established with MSCs and with the fibers (Figure 6B). Briefly, VITVO50 was loaded with 1 × 10^6^ MSCs. After 1 day, VITVO50 was perfused with 2.5 × 10^5^ of TC71 Luc cells for 20 min. After 3 h, gDNA was then extracted and 3-plex ddPCR assay was performed. On average, the VITVO50 were colonized by 82.2% ± 8.9 of MSCs, with a retention of 15.9 ± 5.8% tumor cells. The average tumor cell to MSCs ratio was 0.2 ± 0.1. Collectively, the VITVO50-based fluidic circuit appeared to be a useful tool to analyze interactions that occur between migrating tumor cells and a stromal microenvironment of interest. For future developments, this model promises to easily mimic the dissemination of metastatic cells via the bloodstream to finally colonize the stromal environment of a secondary organ. Again, the 3-plex ddPCR assay proved to be a sensitive and accurate method to quantitatively investigate dynamic cell-to-cell or cell-to-matrix interactions in the VITVO50.

## Discussion

In this study, we developed novel 3D platform-based fluidic models to investigate dynamic cell-to-cell interactions between tumor cells and MSCs, as relevant players in cancer progression as well as promising vehicles of anticancer drugs.

ES is the second most common bone tumor among infants and young adults after osteosarcoma. Despite a marked improvement in the prognosis of patients with localized ES, the mortality caused by metastases and recurrent disease remains unacceptably high, especially when multiple metastatic sites are involved (Choi et al., 2014; Gerrand et al., 2016). At present, an urgent and unmet need exists for the development of novel treatment strategies to improve the outcomes of metastatic ES. In solid tumors, the TME represents a key contributor to cancer progression. Although its role in fostering or suppressing tumor growth needs to be better clarified, TME may represent an interesting target for the development of anticancer therapies (Klopp et al., 2011). The crosstalk between cancer cells and CAFs is complex, and, beyond growth, it can also induce the extravasation and migration of tumor cells towards distant sites (Moskaleva et al., 2019). MSCs are one of the main contributors of the CAF population.

Several 3D technologies have been implemented throughout the years to better understand the physical and molecular interplay among cancer cells and other stromal cells (Rodrigues et al., 2021). The 3D bioreactor called VITVO^Ⓡ^ proved to be an interesting tool for several applications in oncology (Candini et al., 2019). Compared to spheroids, where cells are forced to interact and to form aggregates. in VITVO^Ⓡ^, as in other 3D matrix-supplied devices, cells are free to move through the fibers of the inner core matrix, creating spontaneous cell-to-cell and cell-matrix interplays (Candini et al., 2019). Moreover, the 3D matrix of VITVO^®^ allows cells to grow in a structural supportive environment, where an in vivo-like tumor architecture is more likely to be recreated (Candini et al., 2019).

In the present study, we employed a customized variant of VITVO^Ⓡ^, here named VITVO50. Compared with VITVO^Ⓡ^, VITVO50 is characterized by a wider inter-fiber distance enabling to study interactions between different cell types under dynamic culture conditions. Colonization of the VITVO50 matrix by the tumor was performed connecting the bioreactor to a custom-made fluidic system with cell dose and flow conditions set to obtain a colonization of VITVO50 as homogeneous as possible, while leaving empty spaces in the matrix. Overall, this led to the formation of small in vivo*-*like tumor nodules trapped in the whole thickness of the matrix. Similarly, Fong et al. cultured ES cells on 3D polycaprolactone scaffolds, and studied the effect of fluid perfusion, demonstrating that flow dynamic conditions positively influence tumor cell growth, distribution and gene expression (Fong et al., 2013).

However, the development of dynamic 3D tumor models to study cell-to-cell interaction seems to be a rather unexplored field. Here, interactions between tumor cells and MSCs were investigated in VITVO50. Setting up a VITVO50-based fluidic circuit we were able to model the process of tissue colonization and creation of metastatic nodules by migrating tumor cells, mimicking in a simplified way what happens during tumor dissemination. MSCs were subsequently introduced into the circuit and their interaction with tumor nodules under flow conditions was quantified. A portion of MSCs was effectively trapped into the matrix as a result of active cell-to cell interactions with tumor nodules or to a physical entrapment between the fibers of the matrix.

Our model simulated in a simplified way the process of MSC migration and engraftment into tumor sites. MSCs could sense cancer as a damaged tissue thereby migrating towards it in response to tumor-derived inflammatory molecules, such as SDF-1, TNF-a and interleukins IL-6 and LL-37 (Coffelt et al., 2009; Barcellos-de-Souza et al., 2016). The expression of IL-6 and of TGF-b by tumor cells has a significant role in primary adhesion between MSCs and cancer (Thin Luu et al., 2013). IL-6 and TGF-b are indeed capable of enhancing the expression of integrins on several types of cells, including MSCs, as mediators in cell-to-cell and cell-ECM interplays (Schoeler et al., 2003; Margadant and Sonnenberg, 2010; Zhu et al., 2020). Once MSCs entered the VITVO50, we can speculate that interactions like the integrin-mediated ones took place, allowing few MSCs to engraft into the tumor-colonized matrix. Due to the short interaction time, we hypothesize that interactions occurring in VITVO50 may recall those involved in MSC extravasation. Molecules such as integrins VLA-4, CD106, the cytokine receptor CCL2 and galactin-1, engaged in the process of MSCs extravasation, could take part in the interplay between MSCs and tumor cells in VITVO50 (Belema-Bedada et al., 2008; Melzer et al., 2016; Nitzsche et al., 2017). In future application, the VITVO50-based fluidic circuit could be employed to study tumor-MSC interaction where the MSC affinity for the tumor has been increased by novel targeting strategies (Golinelli et al., 2020). Our group recently optimized tumor-localizing potential of TRAIL MSCs by the expression of an anti-GD2 chimeric antigen receptor (GD2 tCAR) (Golinelli et al., 2020; Golinelli et al., 2021). The disialoganglioside GD2 is a surface molecule expressed at high levels by a wide range of tumors such as osteosarcoma, ES (Heiner et al., 1987; Chang et al., 1992; Kailayangiri et al., 2012; Dobrenkov et al., 2016), neuroblastoma (Prapa et al., 2015) and glioblastoma (Golinelli et al., 2020) and with restricted and low expression in normal tissues (Nazha et al., 2020). The VITVO50-based fluidic circuit could represent a useful tool to easily investigate whether the expression of the GD2 tCAR might improve the MSC affinity for GD2-positive tumors, limiting the use of animal models.

Setting up ddPCR-based assays we were able to quantify colonization and cell-to-cell interaction within the VITVO50 matrix with a high sensitivity. Compared with escape data, ddPCR data showed a higher retention of MSCs in the VITVO50. We demonstrated that during interaction under flow, some tumor cells detached from the matrix, and therefore contributed to the escape. In support, TC71 line is a highly metastatic cell line that by itself grows in semi-adherence conditions and does not establish strong interaction with the support. Despite being more time consuming, ddPCR was far more accurate to study dynamic cell-to-cell interactions in our VITVO50-based fluidic circuit.

Our VITVO50 findings were finally applied for the analysis of archived specimens of a previous experiment conducted *in vivo*. An ES metastatic model was established in NSG mice with lungs and liver as primary organs affected by metastases. Mice were treated with MSCs releasing sTRAIL or MSCs of control. Here, the *in vivo* fate of MSCs was investigated using a 4-plex ddPCR assay, looking at their distribution in mice organs affected by metastases. By increasing the detection capacity using assays with a higher multiplicity, we explored whether ddPCR could precisely quantify the contribution of different cell types in biological samples derived from *in vivo* models, exploring their complexity. Overall, ddPCR data confirmed that MSCs were able to effectively engraft to the lungs and liver of ES model mice. In the sTRAIL group, lung metastases were strongly reduced by the release of sTRAIL compared with control MSC group. However, the number of MSCs engrafted to the lung varied accordingly, maintaining a relatively constant MSC to tumor cell ratio for both groups. Of notice, in the liver, the ratio between sTRAIL MSCs and tumor cell was similar to the one obtained in the VITVO50-based fluidic circuit for the study of interaction between floating MSCs and in vivo-like ES nodules. This indicates that the proposed 3D dynamic model allows to recreate *in vivo*-like conditions, potentially limiting *in vivo* testing.

Our VITVO50-based fluidic circuit was further challenged to mimic the process of tumor dissemination and colonization of the stroma of a secondary organ. VITVO50 was first colonized with MSCs, aiming to recreate a CAF-like stromal microenvironment. Tumor cells were then infused in the circuit and employing a 3-plex ddPCR assay we successfully assessed their migration and retention in the VITVO50, as a result of interactions they established with MSCs and with the matrix. The gold standard for the study of the metastatic process is still represented by *in vivo* models, especially humanized mouse models that incorporate also human-derived stromal components, such as CAFs and immune cell populations. However, *in vivo* models have a high ethical and economic impact (Shultz et al., 2012; Robinson et al., 2019). In this view, the VITVO50-based fluidic system could be molded to mimic the complex *in vivo* microenvironment and address specific questions on the biology of the metastatic process. Importantly, it could be used as a drug screening platform, looking for therapeutic targets to potentially alter the metastatic cascade and guide the preclinical testing of new agents in mouse models (Gómez-Cuadrado et al., 2017; Khan and Steeg, 2018).

In summary, the present work suggests how novel *in vitro* models could facilitate the study of tumor-stroma interaction, simplifying the analysis of *in vivo* data and, ultimately, accelerating the progress towards the early clinical phase. The VITVO50-based fluidic circuit demonstrated to be a manageable and versatile model to develop *in vivo*-like tumor or stromal structures and investigate dynamic cell-to-cell interactions that play a role in cancer progression. ddPCR proved to be a sensitive method to analyze cell colonization and interactions within the dynamic VITVO50 model as well as in samples of an *in vivo* model. The proposed fluidic system promises to be a flexible and further optimizable model to study several biological events, fostering the development of novel therapeutic approaches in the oncology field and beyond.

## Data Availability

The original contributions presented in the study are included in the article/Supplementary Material, further inquiries can be directed to the corresponding authors.
