# Supplementary material for: A 3D Platform to Investigate Dynamic Cell-to-Cell Interactions Between Tumor Cells and Mesenchymal Progenitors
Source: Front Cell Dev Biol. 2022 Jan 17;9:767253. doi: 10.3389/fcell.2021.767253 (PMC8802911; doi:10.3389/fcell.2021.767253)
Supplement: Supplementary file 1 [file DataSheet1.pdf]

**SUPPLEMENTARY TABLE**

| | Primer sequence | Amplified length |
|---|---|---|
| **Luc** | 5'- GCGCCAACTAACGAAAT-3' (FW; sense)<br>5'- CGATAGTAGGTTGGGCTAT-3' (REV; antisense)<br>5’- TGTGGTTCAGCTCTCTAAG-3’ (probe; antisense) | 70 bp |
| **GFP** | 5’- GGACGGCGACGTAAA-3’ (FW; sense)<br>5’- CGTAGGTGGCATCGC -3’ (REV; antisense)<br>5’-CACAAGTTCAGCGTGTC-3’ (probe) | 66 bp |

**Supplementary Table 1.** Custom primers and probes for ddPCR were designed to target selected genes (Luc, GFP).

**SUPPLEMENTARY FIGURE**

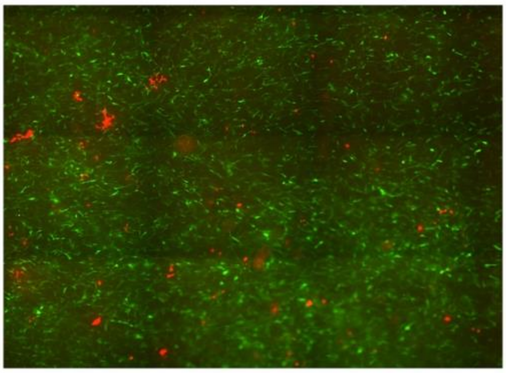

**Figure S1. A small amount of tumor cells escapes from the VITVO50 matrix during interaction under flow with MSCs.** The classic VITVO® was installed downstream of the VITVO50 in the fluidic circuit and used as a sort of sift to trap cells that escaped during interaction, whether they were MSCs or tumor cells. Looking at the scan of nine representative fields of the VITVO® matrix, we observed the presence of few tumor cells (red) that had detached from VITVO50, together with escaped MSCs (green). Objective 4x.
